# Estimating the share of ultra-processed foods in Brazilian municipalities

**DOI:** 10.11606/s1518-8787.2025059006615

**Published:** 2025-05-30

**Authors:** Leandro Teixeira Cacau, Maria Helena D’Aquino Benicio, Renata Bertazzi Levy, Maria Laura da Costa Louzada

**Affiliations:** I Universidade de São Paulo Núcleo de Pesquisas Epidemiológicas em Nutrição e Saúde São Paulo SP Brasil Universidade de São Paulo. Núcleo de Pesquisas Epidemiológicas em Nutrição e Saúde. São Paulo, SP, Brasil; II Universidade de São Paulo Faculdade de Saúde Pública Departamento de Nutrição São Paulo SP Brasil Universidade de São Paulo. Faculdade de Saúde Pública. Departamento de Nutrição. São Paulo, SP, Brasil; III Universidade de São Paulo Faculdade de Medicina Departamento de Medicina Preventiva São Paulo SP Brasil Universidade de São Paulo. Faculdade de Medicina. Departamento de Medicina Preventiva. São Paulo, SP, Brasil

**Keywords:** Ultra-Processed Foods, Socioeconomic Factors, Prediction Models, Epidemiology

## Abstract

**OBJECTIVE:**

To estimate the caloric share of ultra-processed foods (% UPF) in the 5,570 Brazilian municipalities.

**METHODS:**

The estimation of % UPF in municipalities was performed using a statistical prediction model based on data from 46,164 individuals aged over >10 years who participated in the Household Budget Survey (HBS 2017–2018). Multiple linear regression was used to estimate the average % UPF (measured through two 24-hour dietary recalls) based on predictor variables (sex, age, income, education, race/color, urbanity, federative units, and geographic location). The model's adequacy was assessed through residual analysis and by comparing predicted values with those directly measured in POF 2017–2018 using Lin's concordance correlation coefficient (CCC). The linear coefficients obtained from the multiple linear regression model were applied to the sociodemographic data from the 2010 Census (measured similarly to POF) to estimate the % UPF for each municipality.

**RESULTS:**

The statistical model proved adequate, showing normally distributed residuals and a CCC of 0.87, indicating almost perfect agreement. There was heterogeneity in the distribution of % UPF estimates, ranging from 5.75% in Aroeiras do Itaim (PI) to 30.5% in Florianópolis (SC). % UPF estimates were higher (>20%) in municipalities from the South region and the state of São Paulo. Capitals had higher estimates of caloric contribution from ultra-processed foods compared to other municipalities in their states.

**CONCLUSIONS:**

The predictive model revealed differences in % UPF among Brazilian municipalities. The generated estimates can contribute to monitoring ultra-processed food consumption at the municipal level and support the development of public policies focused on promoting healthy eating.

## INTRODUCTION

Ultra-processed foods, as defined by the Nova Food classification, are typically ready-to-eat industrial formulations made with numerous processing steps and ingredients obtained from high-yield plants, such as sugars and syrups, refined starches, protein isolates, as well as animal tissues of low commercial value. These formulations are designed to be visually appealing and have intense or even "irresistible" flavors, using combinations of flavorings, colorings, emulsifiers, sweeteners, and other cosmetic additives^1^.

Consistent evidence has shown that high consumption of ultra-processed foods is associated with diets with nutritionally unbalanced profiles and the presence of potentially toxic components generated during processing^2^ or released from synthetic packaging^3^, excessive energy intake, and insufficient consumption of a variety of fresh or minimally processed foods^4^. In addition, meta-analyses have shown their association with an increased risk of several chronic diseases, such as obesity, diabetes, and cardiovascular diseases^5^. More recently, a review of the literature has also identified positive links between the production of ultra-processed foods and environmental degradation, including loss of biodiversity, high greenhouse gas emissions, inappropriate disposal of packaging, soil degradation, and decreased water quality^6^.

Corroborating this evidence, the Dietary Guidelines for the Brazilian Population recommends, as a golden rule, always preferring fresh or minimally processed foods and culinary preparations to ultra-processed foods^7^. In Brazil, data from the 2017-2018 Household Budget Survey (HBS) showed that ultra-processed foods already account for around 20% of the total calories consumed by Brazilians. This consumption is higher among women and in the South and Southeast regions, and lower among black and brown people and those living in rural areas, as well as decreasing with age and increasing with education and income^8^.

Despite the representativeness of the Brazilian population offered by the HBS, it does not allow estimates to be broken down to the level of municipalities due to its sample planning. This gap prevents the identification of intra-regional inequalities, and the promotion and monitoring of public policies geared to the reality of each municipality. The aim of this study was to estimate the calorie intake of ultra-processed foods in the 5,570 Brazilian municipalities.

## METHODS

This is a study based on individual food consumption and sociodemographic data from participants in the most recent POF, carried out in 2017–2018, and sociodemographic information from Brazilian municipalities extracted from the 2010 Census sample. The method used to obtain estimates of the calorie intake of ultra-processed foods in Brazilian municipalities was based on the development of a statistical prediction model, which consists of an equation that allows the average value of a certain outcome to be estimated as a function of the characteristics of the predictor variables.

### Construction of a Model to Predict the Caloric Share of Ultra-Processed Foods

The empirical basis for building the predictive model was the sample of 46,164 individuals aged >10 years who took part in the individual food consumption module of the 2017–2018 POF. The POF sample follows the parameters of complex samples, considering the country's social and geographical strata, making it representative of the Brazilian population aged >10 years for Brazil, according to major regions, in urban and rural areas, in the Federation Units (FU), and in the 26 capitals and the Federal District (DF)^9^.

In the 2017–2018 POF, food consumption was assessed using two 24-hour dietary recalls (R24h) on non-consecutive days^10^. More than 1,800 foods were reported in the two 24hRs, which were classified, according to Nova, into: i) fresh or minimally processed foods; ii) processed culinary ingredients; iii) processed foods; and iv) ultra-processed foods. The Brazilian Food Composition Table was used to obtain information on the amount of energy (kcal) in each food and to calculate the total energy intake of each individual.

The caloric share (% of total energy consumed) of ultra-processed foods was the model's response variable, calculated from the average of the two 24hR. The predictor variables were based on the model of factors associated with the consumption of ultra-processed foods proposed by Louzada et al^8^. and the availability and compatibility of the information collected by the 2017–2018 POF and the 2010 Census sample. The variables and their respective categories were: sex (male/female), age group (10–14, 15–19, 20–24, 25–29, 30–34, 35–39, 40–44, 45–49, 50–54, 55–59, 60–64, 65–69, and >70 years), self-reported race/skin color (white, black, yellow, brown, indigenous, or non-respondents), education level (up to incomplete primary education, complete primary education to incomplete secondary education, complete secondary education to incomplete higher education, and complete higher education), *per capita* household income (≤ 1/4, > 1/4–1/2, > 1/2–1, > 1–2, > 2–3, > 3–5, and > 5 minimum wages - MW) and household situation (urban/rural). In addition, FU were included, with 27 categories, and a categorical variable that differentiates between capitals, cities in metropolitan regions and other municipalities.

A multiple linear regression model was used, including all the predictor variables simultaneously in the model. The statistical significance of each variable included in the model was assessed using the t-test. A significance level of < 0.05 was adopted. The final model was assessed for its validity using two analyses. Firstly, residual analysis was carried out, which checks whether the model's residuals are normally distributed around zero, ensuring that the model adequately captures the relationship between the predictor variables and the response variable. Secondly, we went on to assess the agreement between the average calorie intake of ultra-processed foods predicted by the model and that detected directly in the 2017/2018 POF in the 26 Brazilian state capitals and the Federal District, using Lin's correlation-concordance coefficient (CCC). The CCC determines how far the observed data deviates from the line of perfect agreement, i.e. the 45° line on a scatter plot. To interpret the coefficients, we consider: 0.00-0.20 slight agreement; 0.21-0.40 fair agreement; 0.41-0.60 moderate agreement; 0.61-0.80 substantial agreement; 0.81-1.00 almost perfect agreement; and 1 perfect agreement^11^.

### Generation of Estimates of the Caloric Share of Ultra-Processed Foods in Municipalities

Estimates of the calorie share of ultra-processed foods for each municipality were obtained by applying the model equation (constant and linear coefficients β for each predictor variable) to the sociodemographic data for each of the municipalities in the 2010 Census sample, considering only residents aged > 10 years. The questionnaire applied to households allows data to be obtained on the distribution of the sociodemographic variables measured in a similar way to that used by the 2017-2018 POF. The planning of the 2010 Census sample guarantees population representativeness of each Brazilian municipality through systematic sampling within each census sector.

For example, to estimate the calorie share of ultra-processed foods in hypothetical municipality A, we used the values of the constant and the coefficients obtained in the regression and multiplied the coefficients by the percentages of the categories of each of the predictor variables. Then the products were added up and the estimated consumption of ultra-processed foods in municipality A was obtained.


$$\text{\%}estimated\;UPF=constant\;+\left(\beta^{*}\text{\%}of\;women\;\right)+\left(\beta^{*}\text{\%}from\;15\;to\;19\;years\;old\;\right)+\ldots$$

UPF = ultra-processed foods.

β = linear coefficient

Considering the changes in the sociodemographic profile of the Brazilian population between 2010 and 2018, such as the increase in income and schooling, it was to be expected that there would be differences between the values directly measured by the 2017-2018 POF and the estimates generated from the variables in the 2010 Census sample. To correct this difference, correction factors were applied to the estimates for each of the 5,570 municipalities. These factors were calculated using the ratio between the average detected directly by the 2017–2018 POF and the value estimated from the 2010 Census sample for each of the Brazilian capitals and the Federal District. The correction factor for each capital and the Federal District was then applied to all the municipalities in its respective state, assuming a homogeneous difference within the state. The Brazilian capitals and the Federal District are the only ones that, individually, have population representativeness guaranteed by the POF sample, making this comparison and generation of the correction factor possible.

After the 2010 Census, Brazil created five municipalities which do not have socioeconomic data available: Balneário Rincão (SC), Mojuí dos Campos (PA), Pinto Bandeira (RS), Paraíso das Águas (MS), and Pescaria Brava (SC). For these, we assumed the estimated calorie share of ultra-processed foods of the municipalities to which they belonged in 2010, namely: Içara (SC), Santarém (PA), Bento Gonçalves (RS), Costa Rica (MS), and Laguna (SC), respectively.

Statistical analyses were carried out using Stata 16.1 software. The estimates of the caloric share of ultra-processed foods were presented in a heat map. The shade of the graph increases progressively with the increase in the ultra-processed food share category. QGIS software and Digital Municipal Mesh of Brazil were used to create the map.

## RESULTS

Table 1 shows the distribution (frequencies) of sociodemographic variables and the average share of ultra-processed foods in the total calories consumed by the Brazilian population aged >10 years, according to the 2017–2018 POF.

**Table 1:** Share of ultra-processed foods in total calories consumed by the Brazilian population aged >10 and over, according to sociodemographic variables. 2017–2018 POF.

Characteristics		%	% calorie share of ultra-processed foods		
			Average	95%CI	
Brazil		100	20.2	19.8	20.6
Sex					
	Male	47.9	19.6	19.1	20.1
	Female	52.1	20.6	20.2	21.1
Age group (in years)					
	10–14	8.5	27.5	26.7	28.4
	15–19	9.3	26.2	24.7	27.7
	20–24	8.5	24.1	23.1	25.2
	25–29	8.38	22.9	21.8	24.0
	30–34	8.56	20.5	19.5	21.5
	35–39	9.1	19.6	18.6	20.7
	40–44	8.0	17.5	16.7	18.3
	45–49	7.7	18.3	17.3	19.2
	50–54	7.5	16.8	15.9	17.6
	55–59	6.7	15.9	15.2	16.6
	60–64	5.6	15.3	14.6	16.0
	65–69	4.3	15.8	14.8	16.8
	> 70	7.7	15.3	14.6	16.0
Self-declared race/color					
	White	43.1	21.9	21.4	22.4
	Black	10.8	18.8	17.8	19.8
	Yellow	0.7	23.5	20.1	26.8
	Brown	44.9	18.8	18.2	19.3
	Indigenous	0.4	21.5	17.6	25.5
	No declaration	0.1	25.7	13.4	38.0
Education					
	Up to incomplete primary education	41.9	17.2	16.8	17.5
	Complete primary education to incomplete secondary education	16.8	20.7	19.9	21.4
	Complete secondary education to incomplete higher education	30.7	22.5	21.7	23.3
	Complete higher education	10.6	24.3	23.4	25.2
*Per capita* income (minimum wage)					
	≤ 1/4	4.9	15.8	14.7	16.9
	> 1/4–½	12.3	16.8	16.0	17.6
	> 1/2–1	24.3	18.4	17.9	19.0
	> 1–2	31.6	20.8	19.9	21.8
	> 2–3	11.8	21.8	29.9	22.7
	> 3–5	8.2	23.1	22.0	24.2
	> 5	6.9	25.9	24.5	27.4
Home situation					
	Urban	85.5	21.2	20.7	21.7
	Rural	14.5	14.1	13.6	14.5
Municipal situation					
	Capital	23.9	23.0	21.7	24.2
	Metropolitan Region	15.3	22.0	21.0	23.0
	Others	60.8	18.6	18.2	19.0
Federation Unit					
	Rondônia	0.8	14.2	12.9	15.4
	Acre	0.4	16.7	14.7	18.6
	Amazonas	1.8	16.8	15.7	18.0
	Roraima	0.2	16.7	15.0	18.4
	Pará	3.9	18.8	17.4	20.3
	Amapá	0.4	21.3	19.7	22.8
	Tocantins	0.7	12.9	11.1	14.7
	Maranhão	3.3	13.1	12.1	14.0
	Piauí	1.6	11.5	10.8	12.3
	Ceará	4.3	17.8	16.6	19.0
	Rio Grande do Norte	1.7	18.7	17.6	19.9
	Paraíba	0.2	16.1	15.0	17.2
	Pernambuco	0.5	19.3	18.4	20.2
	Alagoas	1.6	16.6	15.4	17.9
	Sergipe	0.1	18.4	16.9	19.9
	Bahia	7.1	19.4	18.4	20.4
	Minas Gerais	10.2	19.5	18.6	20.3
	Espírito Santo	1.9	16.4	15.3	17.4
	Rio de Janeiro	8.4	21.1	20.1	22.2
	São Paulo	22.1	23.5	22.0	25.0
	Paraná	5.5	20.7	19.4	22.1
	Santa Catarina	3.4	23.9	22.6	25.1
	Rio Grande do Sul	5.6	24.8	23.7	26.0
	Mato Grosso do Sul	1.3	19.6	18.3	20.9
	Mato Grosso	0.2	16.7	15.3	18.1
	Goiás	3.4	17.4	16.2	18.6
	Distrito Federal	1.4	22.5	21.0	23.9

In 2017-2018, the frequency of women was slightly higher than that of men (52.1% versus 47.9%), 17.8% were adolescents and 17.6% were elderly. The brown race/color concentrated the largest fraction of the population (44.9%) and 41.9% had up to incomplete primary schooling. The majority (55.9%) of the population had a *per capita* income of between half and two minimum wages. Around 85% lived in urban areas and most lived in the state of São Paulo (22.1%).

In 2017-2018, the average calorie intake of ultra-processed foods in Brazil was 20.2% (95% confidence intervals - 95%CI 19.8–20.6), with variation according to sociodemographic characteristics. The average calorie intake was slightly higher in women compared to men, decreased with increasing age, and increased according to education level and income. The average calorie intake was higher among urban dwellers compared to rural dwellers, and in capital cities compared to other municipalities. Average calorie intake was higher in the Federal District and in the southern and southeastern states, except for Espírito Santo. On the other hand, the states in the North and Northeast, apart from Bahia, had the lowest averages (Table 1).

### Model for Predicting the Caloric Share of Ultra-Processed Foods

Table 2 shows the β coefficients and their respective 95%CI of the multiple linear regression model, which tests the association between the predictor variables and the calorie share of ultra-processed foods in the total calories consumed by the Brazilian population aged >10 years. All the predictor variables were statistically associated with the outcome (p < 0.001). The predictor variables with the highest β coefficients and therefore the greatest explanatory power were *per capita* family income and age. Individuals with a *per capita* family income of > 5 minimum wages had an average calorie intake of ultra-processed foods that was 8.4 percentage points (p.p.) higher than those with a per capita family income of less than or equal to 1/4 minimum wage. Individuals aged 70 and over had an average calorie intake from ultra-processed foods that was 15.8 percentage points lower than those aged between 10 and 14.

**Table 2:** β coefficients and their respective 95% confidence intervals of the linear regression model, associating the predictor variables and the calorie share of ultra-processed foods in the total calories consumed by the Brazilian population aged >10 years and over. 2017–2018 POF.

Characteristics		% calorie share of ultra-processed foods			
		β	95%CI		p-value
Sex					< 0,001
	Male	Ref.			
	Female	1.3	0,9	1,7	
Age group (in years)					< 0,001
	10–14	Ref.			
	15–19	-2.9	-4,4	-1,4	
	20–24	-6.5	-7,8	-5,1	
	25–29	-8.4	-9,7	-7,1	
	30–34	-10.5	-11,6	-9,4	
	35–39	-11.4	-12,5	-10,3	
	40–44	-13.2	-14,3	-12,1	
	45–49	-12.8	-13,9	-11,6	
	50–54	-14.4	-15,5	-13,3	
	55–59	-15.5	-16,5	-14,4	
	60–64	-16.2	-17,2	-15,1	
	65–69	-15.5	-16,7	-14,2	
	> 70	-15.8	-16,9	-14,7	
Self-declared race/color					< 0,001
	White	Ref.			
	Black	-0.9	-2,1	0,2	
	Yellow	0.3	-2,4	3,0	
	Brown	-0.8	-1,5	0,0	
	Indigenous	1.7	-1,7	5,1	
	No declaration	4.4	-5,5	14,4	
Education					0,002
	Up to incomplete primary education	Ref.			
	Complete primary education to incomplete secondary education	0.8	0,0	1,5	
	Complete secondary education to incomplete higher education	2.8	2,1	3,6	
	Complete higher education	3.6	2,5	4,6	
*Per capita* income (minimum wage)					< 0,001
	≤ 1/4	Ref.			
	> 1/4–1/2	0.9	-0,4	2,2	
	> 1/2–1	2.6	1,3	3,8	
	> 1–2	5.0	3,7	6,4	
	> 2–3	5.6	4,2	7,0	
	> 3–5	6.2	4,5	7,9	
	> 5	8.4	6,4	10,3	
Home situation					< 0,001
	Urban	Ref.			
	Rural	-3.4	-4,1	-2,8	
	Municipal situation				< 0,001
	Capital	Ref.			
	Metropolitan Region	-1.5	-3,1	0,2	
	Others	-2.4	-3,8	-1,1	
Federation Unit					< 0,001
	Rondônia	Ref.			
	Acre	1.2	-1,1	3,4	
	Amazonas	0.9	-0,9	2,7	
	Roraima	0.3	-2,2	2,7	
	Pará	5.1	3,1	7,1	
	Amapá	5.0	3,0	7,1	
	Tocantins	-1.0	-3,2	1,2	
	Maranhão	0.3	-1,3	1,8	
	Piauí	-1.4	-3,0	0,1	
	Ceará	4.0	2,3	5,7	
	Rio Grande do Norte	4.4	2,7	6,1	
	Paraíba	2.9	1,2	4,6	
	Pernambuco	5.2	3,6	6,8	
	Alagoas	3.3	1,5	5,0	
	Sergipe	3.6	1,8	5,4	
	Bahia	5.7	4,1	7,3	
	Minas Gerais	4.8	3,3	6,4	
	Espírito Santo	1.6	-0,2	3,3	
	Rio de Janeiro	5.0	3,3	6,7	
	São Paulo	7.0	4,9	9,1	
	Paraná	5.1	3,3	6,9	
	Santa Catarina	8.5	6,6	10,4	
	Rio Grande do Sul	9.3	7,5	11,1	
	Mato Grosso do Sul	3.4	1,5	5,2	
	Mato Grosso	1.4	-0,5	3,2	
	Goiás	1.7	-0,1	3,5	
	Distrito Federal	3.2	1,2	5,3	

**Figure 1:**
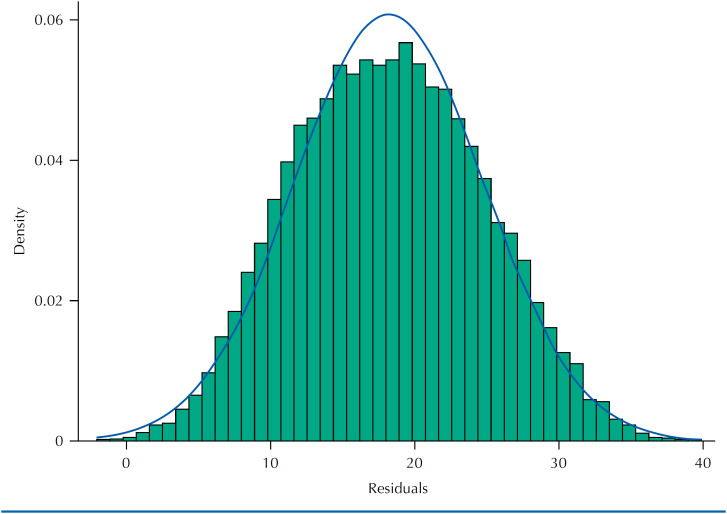
Distribution of residuals from the multiple linear regression model associating the predictor variables and the calorie share of ultra-processed foods in the total calories consumed by the Brazilian population aged >10 and over. The normal distribution indicates a valid model. 2017/2018 POF.

**Figure 2:**
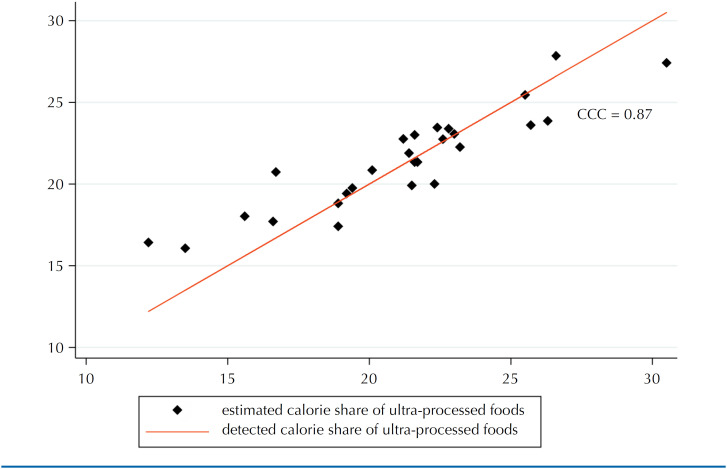
Concordance between the average calorie share of ultra-processed foods predicted by the adjusted model and that detected directly in the 2017/2018 POF. CCC, Lin's correlation-concordance coefficient.

The residuals of the model showed a distribution close to normal (Figure 1), indicating its validity in adequately capturing the relationship between the predictor variables and the outcome. In addition, the agreement between the average calorie intake of ultra-processed foods predicted by the model and that detected directly in the 2017/2018 HBS for the 26 Brazilian state capitals and the Federal District was considered satisfactory, with a CCC of 0.87 (almost perfect agreement) (Figure 2).

### Estimates of the Calorie Content of Ultra-Processed Foods in Brazil's 5,570 Municipalities

**Figure 3:**
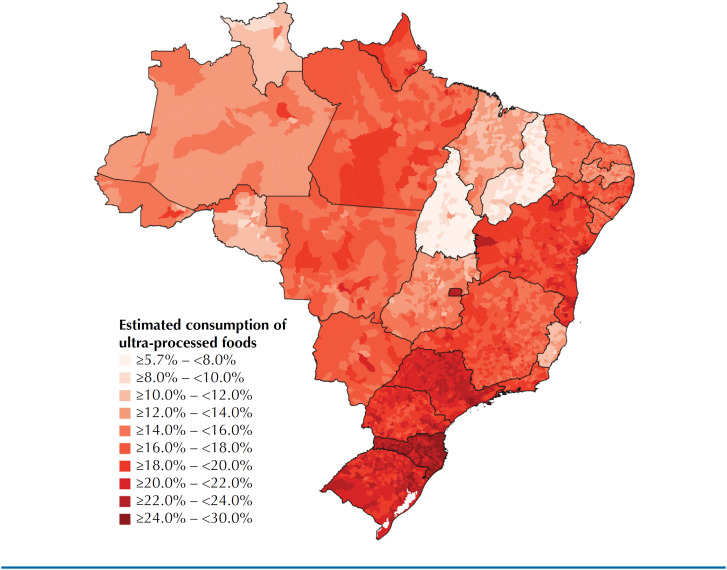
Spatial distribution of estimates of the Caloric share of ultra-processed foods in Brazilian municipalities^a^.

The estimates of the calorie share of ultra-processed foods for each of the 5,570 municipalities are illustrated in the heat map (Figure 3)^a^. Heterogeneity was observed in the estimates of the calorie share of ultra-processed foods in the country. The range covered municipalities with a calorie share as low as around 6% (5.75% in Aroeiras do Itaim, Piauí; 5.83% in Dois Irmãos do Tocantins and 5.87% in Monte Santo do Tocantins, both in Tocantins) and as high as around 30% (27.8% in Balneário Camboriú, 28.3% in São José, and 30.5% in Florianópolis, all in Santa Catarina). The capitals have higher estimates of the share of calories from ultra-processed foods than the other municipalities in their respective states.

Estimates of the calorie share of ultra-processed foods were higher in municipalities in the South, especially in Santa Catarina, where all municipalities had estimates above 20%. In Paraná, estimates ranged from 16% in the municipality of Antônio Olinto to 26.3% in Curitiba. In Rio Grande do Sul, estimates ranged from 17.6% in the municipality of Coqueiro Baixo to 26.6% in Porto Alegre.

In the Southeast, municipalities in the state of São Paulo had the highest estimates, with the capital reaching 25.5%. On the other hand, municipalities in the state of Espírito Santo had the lowest estimates in the region, with the capital Vitória having 16.7%. The state of Minas Gerais stood out as one of the states with the most heterogeneous municipal estimates in the region, with Frei Lagonegro reaching 13.8% and, at the other extreme, the municipalities of Nova Lima and Belo Horizonte reaching 20.9% and 22.8%, respectively. Municipalities in the state of Rio de Janeiro had estimates ranging from 15.2% for São José de Ubá to 22.4% in the capital.

In the Central-West, the municipalities of Goiás had the lowest estimates in the region, with the highest recorded in the capital Goiânia (19.4%). Brasília was the municipality with the highest average calorie intake from ultra-processed foods in the region, at 22.6%. Mato Grosso stood out as the state with the greatest heterogeneity in the estimates of the region's municipalities, ranging from 13.0% in Barão de Melgaço and Porto Estrela to around 18% in Campo Novo dos Parecis, Várzea Grande, and Cuiabá.

In the Northeast, the states whose municipalities had the lowest estimates were, in ascending order, Piauí, Maranhão and Paraíba. The state of Bahia stood out for having municipalities with the highest estimates in the region, with all of them above 16.0% and the capital Salvador reaching 25.7%.

The municipalities in the state of Tocantins had the lowest estimates of calorie intake from ultra-processed foods in the North, with the highest being in the capital Palmas, which reached 12.2%. The states of Roraima and Rondônia had intermediate estimates in the region. The municipality of Cantá, in Roraima, had an estimate of 9.25% and the municipality of Novo Horizonte do Oeste, in Rondônia, 9.46%, while the capitals had estimates of 15.6% and 16.6%, respectively. The municipalities in the states of Pará and Amapá had the highest estimates in the region.

## DISCUSSION

This study, using a statistical prediction model built from representative data from a food survey, estimated the calorie share of ultra-processed foods in the 5,570 Brazilian municipalities, which ranged from 5.7% to 30.5%. The prediction model was considered valid for estimating the calorie share of ultra-processed foods.

The wide variation in the estimates obtained in this study reflects the unequal distribution of sociodemographic characteristics between municipalities. For example, all the municipalities with more than 15.0% of the population with a *per capita* income of five minimum wages or more had estimates of the calorie intake of ultra-processed foods above 20.0%, except for Vitória (ES). On the other hand, most municipalities in which 0% of the population have a per capita income of more than five minimum wages had estimates well below this value.

Moreover, having a higher proportion of people living in urban areas also proved to be a determining factor in the estimated calorie intake of ultra-processed foods in Brazilian municipalities. The states of Maranhão, Piauí, and Tocantins, which have the highest proportions of people living in rural areas in the country, had the lowest estimates of calorie intake from ultra-processed foods. On the other hand, São Paulo, one of the states with the highest proportion of urban dwellers in the country, had municipal estimates above 18.0%. In addition, all 26 state capitals and the Federal District had higher estimates compared to the other municipalities in their respective states.

It's important to note that even if it's positive that some municipalities have low estimates of calorie intake from ultra-processed foods, this doesn't necessarily imply better performance in other indicators of diet quality. There is evidence that low-income households in rural regions of the country, for example, have a high consumption of fresh or minimally processed foods and culinary ingredients, but that this consumption is marked by staple foods such as rice, beans, coffee, sugar, flour, and meat, and that the consumption of other healthy foods such as fruit, legumes, and vegetables, as well as dietary diversity, is much lower than recommended^12^.

A previous study using household food availability data showed that the purchase of ultra-processed foods increased from 14.3% of total calories in 2002-2003 to 19.4% in 2017-2018^13^. Similarly, Louzada et al.^8^, using individual food consumption data, observed an increase in the actual consumption of ultra-processed foods between 2008 and 2009, and 2017 and 2018. It is clear that the increase in consumption of ultra-processed foods was more pronounced in the North and Northeast regions of the country, as well as in rural regions and those with lower *per capita* incomes. In this way, the results indicate that those municipalities with the lowest estimated consumption in this study are most likely those that are also showing a greater increase in their consumption, indicating that the estimates may be different when a new POF is updated.

Estimating the calorie share of ultra-processed foods is relevant and can support various initiatives, such as investigating environmental factors related to the production, supply, and availability of ultra-processed foods that influence the local reality, developing specific and targeted public policies, and monitoring the impact of these policies by continually making estimates based on future POFs. Such actions can be fundamental to promoting healthier and more sustainable eating in a more equitable way.

Municipal adherence to the National Food and Nutrition Security System (Sisan) allows access to federal government resources for policies and programs at the municipal level, such as the Solidarity Kitchens Program, which aims to provide access to healthy food, respecting the precepts of the Dietary Guidelines for the Brazilian Population. Another example is the Popular Restaurant Program, a social facility that is part of Sisan's operational structure, which aims to expand the supply of nutritionally adequate meals at affordable prices for socially vulnerable populations. In the state of São Paulo, the Bom Prato program stands out as one of the largest in the country. Local actions, such as the municipal laws in Rio de Janeiro and Niterói banning the sale of ultra-processed foods in public and private school canteens, are examples of initiatives aimed at reducing the consumption of these products among children and adolescents^14,15^.

Recently, the Ministry of Social Development and Fight Against Hunger launched a strategic plan that identifies 60 priority cities for the implementation of the National Strategy for Food and Nutritional Security in Cities, called *Alimenta Cidades*^16^. This initiative seeks to increase production, access, availability, and consumption of healthy food for socially vulnerable populations. Among the prioritized cities, all the country's capitals stand out, as they showed high estimates of consumption of ultra-processed foods compared to the other municipalities in their states.

The study's main limitation is the time difference between the 2017-2018 HBS and the 2010 Population Census. Although a new Census was carried out in 2022, its data (such as income) is not available for consultation. However, to mitigate this limitation, correction factors for each of the 26 state capitals and the Federal District were calculated and applied to the municipalities in their respective states. In addition, the food consumption module of the 2017-2018 POF did not include people aged >10 years, which means that the results of this study only apply to the Brazilian population of adolescents and adults. As strengths, we can mention that the prediction model created to obtain estimates of consumption of ultra-processed foods in the municipalities showed satisfactory results of adequacy, with a residual with a distribution close to normal and high agreement between the average calorie intake predicted by the model and that detected directly in the 2017-2018 HBS for the capitals and the Federal District. In addition, the study, in a pioneering way, generated municipal estimates of the calorie share of ultra-processed foods, valuable information for municipal managers.

In conclusion, this study generated estimates of the calorie intake of ultra-processed foods in Brazilian municipalities. It was possible to observe heterogeneity in the municipal estimates, which reflects the sociodemographic inequality of the Brazilian population. The estimates generated can contribute to monitoring food consumption of ultra-processed foods at the municipal level and strengthen and subsidize the creation of public policies focused on promoting healthy eating.

## Footnotes

a Also available at: https://doi.org/10.17605/OSF.IO/ZM9CR.
