# Supplementary material for: Estimating the share of ultra-processed foods in Brazilian municipalities
Source: Rev Saude Publica. 2025 May 30;59:e22. doi: 10.11606/s1518-8787.2025059006615 (PMC12207894; doi:10.11606/s1518-8787.2025059006615)
Supplement: Translation

# Translation: Estimativa da participação de alimentos ultraprocessados nos municípios brasileiros

**Authors:** Leandro Teixeira Cacau, Maria Helena D’Aquino Benicio, Renata Bertazzi Levy, Maria Laura da Costa Louzada

**Affiliations:** I São Paulo SP Brasil Universidade de São Paulo. Núcleo de Pesquisas Epidemiológicas em Nutrição e Saúde. São Paulo, SP, Brasil; II São Paulo SP Brasil Universidade de São Paulo. Faculdade de Saúde Pública. Departamento de Nutrição. São Paulo, SP, Brasil; III São Paulo SP Brasil Universidade de São Paulo. Faculdade de Medicina. Departamento de Medicina Preventiva. São Paulo, SP, Brasil

**Keywords:** Alimentos Ultraprocessados, Fatores Socioeconômicos, Modelos de Predição, Epidemiologia

## Abstract

**OBJETIVO:**

Estimar a participação calórica de alimentos ultraprocessados (% AUP) nos 5.570 municípios brasileiros.

**MÉTODOS:**

A estimativa de % AUP nos municípios foi realizada a partir de um modelo estatístico de predição construído com base nos dados de 46.164 indivíduos com idades >10 anos participantes da Pesquisa de Orçamentos Familiares (POF 2017–2018). Regressão linear múltipla foi usada para estimar a % média de AUP (medida por meio de dois recordatórios alimentares de 24 h) em função das variáveis preditoras (sexo, idade, renda, escolaridade, raça/cor, urbanidade, Unidades da Federação e localização geográfica). O modelo foi avaliado quanto a sua adequabilidade por meio de análise de resíduos e pela comparação entre valores preditos pelo modelo e mensurados diretamente na POF 2017–2018 por meio do coeficiente de correlação-concordância de Lin (CCC). Os coeficientes lineares obtidos no modelo de regressão linear múltiplo foram aplicados aos dados sociodemográficos da amostra do Censo de 2010 (aferidos de forma semelhante à da POF) para estimar o % de AUP de cada um dos municípios.

**RESULTADOS:**

O modelo estatístico mostrou-se adequado, apresentando distribuição normal dos resíduos e um CCC de 0,87, indicando concordância quase perfeita. Foi observada uma heterogeneidade na distribuição das estimativas de % AUP, variando de 5,75% em Aroeiras do Itaim (PI) a 30,5% em Florianópolis (SC). As estimativas de % AUP foram mais altas (>20%) em municípios da região Sul e do estado de São Paulo. As capitais apresentam maiores estimativas de participação calórica de alimentos ultraprocessados em relação aos demais municípios de seus estados.

**CONCLUSÕES:**

O modelo preditivo revelou diferenças de % AUP entre os municípios brasileiros. As estimativas geradas podem contribuir para o monitoramento do consumo alimentar de ultraprocessados no nível municipal e fortalecer e subsidiar a criação de políticas públicas focadas na promoção da alimentação saudável.

## INTRODUÇÃO

Alimentos ultraprocessados, como definidos pela classificação de alimentos Nova, são formulações industriais tipicamente prontas para consumo feitas em inúmeras etapas de processamento e ingredientes obtidos a partir de plantas de alto rendimento, como açúcares e xaropes, amidos refinados, isolados proteicos, além de tecidos de animais de baixo valor comercial. Essas formulações são feitas para serem visualmente atrativas e terem sabores intensos ou mesmo "irresistíveis", usando para isso combinações de flavorizantes, corantes, emulsificantes, edulcorantes e outros aditivos de natureza cosmética^1^.

Evidências consistentes têm demonstrado que o elevado consumo de alimentos ultraprocessados está associado a dietas com perfis nutricionalmente desbalanceados e com presença de componentes potencialmente tóxicos gerados durante o processamento^2^ ou liberados de embalagens sintéticas^3^, excessiva ingestão de energia e consumo insuficiente de uma diversidade de alimentos *in natura* ou minimamente processados^4^. Além disso, meta-análises têm demonstrado sua associação com um maior risco de uma série de doenças crônicas, como, por exemplo, obesidade, diabetes e doenças cardiovasculares^5^. Mais recentemente, uma revisão da literatura também identificou relações positivas entre a produção de alimentos ultraprocessados e a degradação ambiental, incluindo perda de biodiversidade, emissões elevadas de gases de efeito estufa, a disposição inadequada de embalagens, a degradação do solo e a diminuição da qualidade da água^6^.

Corroborando essas evidências, o Guia Alimentar para a População Brasileira recomenda, como regra de ouro, que se prefira sempre alimentos *in natura* ou minimamente processados e preparações culinárias a alimentos ultraprocessados^7^. No Brasil, dados da Pesquisa de Orçamentos Familiares (POF) de 2017-2018 mostraram que os alimentos ultraprocessados já representam cerca de 20% do total de calorias consumidas pelos brasileiros. Esse consumo é maior no sexo feminino e nas regiões Sul e Sudeste e menor em pessoas pretas e pardas e moradoras da área rural, além de diminuir com o aumento da idade e aumentar com escolaridade e renda^8^.

Apesar da representatividade da população brasileira oferecida pela POF, ela não permite desagregações de estimativas para o nível de municípios em face de seu planejamento amostral. Essa lacuna impede a identificação de desigualdades intrarregionais e impulsionamento e monitoramento de políticas públicas direcionadas para a realidade de cada município. O objetivo deste trabalho foi estimar a participação calórica de alimentos ultraprocessados nos 5.570 municípios brasileiros.

## MÉTODOS

Trata-se de um estudo realizado a partir dos dados de consumo alimentar individual e sociodemográficos dos participantes da mais recente POF, realizada em 2017-2018, e das informações sociodemográficas dos municípios brasileiros extraídas da amostra do Censo Demográfico de 2010. O método empregado para obtenção das estimativas de participação calórica de alimentos ultraprocessados nos municípios brasileiros baseou-se no desenvolvimento de um modelo estatístico de predição, que consiste em uma equação que permite estimar o valor médio de um certo desfecho em função das características das variáveis preditoras.

### Construção do Modelo de Predição da Participação Calórica de Alimentos Ultraprocessados

Utilizou-se como base empírica para a construção do modelo preditivo a amostra de 46.164 indivíduos com >10 anos de idade, participantes do módulo de consumo alimentar individual da POF 2017–2018. A amostra da POF segue os parâmetros de amostras complexas, considerando estratos sociais e geográficos do país, tornando-se, desta forma, representativo da população brasileira >10 anos de idade para o Brasil, segundo grandes regiões, em áreas urbanas e rurais, nas Unidades da Federação (UF) e nas 26 capitais e no Distrito Federal (DF)^9^.

Na POF 2017–2018, o consumo alimentar foi avaliado por meio da aplicação de dois recordatórios alimentares de 24 horas (R24h) em dias não consecutivos^10^. Foram reportados mais de 1.800 alimentos nos dois R24h, os quais foram classificados, segundo a Nova, em: i) alimentos *in natura* ou minimamente processados; ii) ingredientes culinários processados; iii) alimentos processados e iv) alimentos ultraprocessados. A Tabela Brasileira de Composição de Alimentos foi utilizada para obtenção das informações sobre a quantidade de energia (kcal) de cada alimento e cálculo do consumo energético total de cada indivíduo.

A participação calórica (% do total de energia consumida) de alimentos ultraprocessados foi a variável resposta do modelo, calculada a partir da média dos dois R24h. As variáveis preditoras foram baseadas no modelo de fatores associados ao consumo de alimentos ultraprocessados proposto por Louzada e colaboradores^8^ e a disponibilidade e compatibilidade das informações coletadas pela POF 2017-2018 e pela amostra do Censo 2010. As variáveis e suas respectivas categorias foram: sexo (homem/mulher), faixa-etária (10–14, 15–19, 20–24, 25–29, 30–34, 35–39, 40–44, 45–49, 50–54, 55–59, 60–64, 65–69 e >70 anos), raça/cor da pele autodeclarada (branca, preta, amarela, parda, indígena ou não respondentes), escolaridade (até o ensino fundamental incompleto, ensino fundamental completo a ensino médio incompleto, ensino médio completo a ensino superior incompleto e ensino superior completo), renda domiciliar *per capita* (≤ 1/4, > 1/4–1/2, > 1/2–1, > 1–2, > 2–3, > 3–5 e > 5 salários mínimos – SM) e situação do domicilio (urbano/rural). Adicionalmente, foram incluídas as UF, com 27 categorias, e uma variável categórica que diferencia capitais, cidades das regiões metropolitanas e demais municípios.

Utilizou-se modelo de regressão linear múltiplo, incluindo todas as variáveis preditoras simultaneamente no modelo. A significância estatística de cada variável incluída no modelo foi avaliada pelo teste t. Adotou-se um nível de significância < 0,05. O modelo final foi avaliado quanto a sua validade por meio de duas análises. Primeiro, realizou-se análise de resíduos, que verifica se os resíduos do modelo se distribuem normalmente ao redor de zero, garantindo que o modelo captura adequadamente a relação entre as variáveis preditoras e a variável resposta. Segundo, prosseguiu-se a avaliação da concordância entre a média da participação calórica de alimentos ultraprocessados predita pelo modelo e a detectada diretamente na POF 2017/2018 nas 26 capitais brasileiras e no DF, através do coeficiente de correlação-concordância (CCC) de Lin. O CCC determina o quão distantes os dados observados se desviam da linha de concordância perfeita, ou seja, a linha a 45° em um gráfico de dispersão. Para interpretação dos coeficientes, consideramos: 0,00–0,20 concordância ligeira; 0,21–0,40 concordância justa; 0,41–0,60 concordância moderada; 0,61–0,80 concordância substancial; 0,81–1,00 concordância quase perfeita e 1, concordância perfeita^11^.

### Geração das Estimativas de Participação Calórica de Alimentos Ultraprocessados nos Municípios

As estimativas de participação calórica de alimentos ultraprocessados para cada município foram obtidas aplicando-se a equação do modelo (constante e coeficientes lineares β para cada variável preditora) nos dados sociodemográficos de cada um dos municípios da amostra do Censo 2010, considerando somente os moradores de >10 anos. O questionário aplicado nos domicílios permite a obtenção de dados relativos à distribuição das variáveis sociodemográficas aferidas de forma semelhante à utilizada pela POF 2017–2018. O planejamento da amostra do Censo 2010 garante representatividade populacional de cada um dos municípios brasileiros por meio de amostragem sistemática dentro de cada setor censitário.

Por exemplo, para estimar a participação calórica de alimentos ultraprocessados no município hipotético A, empregou-se os valores da constante e dos coeficientes obtidos na regressão e multiplicou-se os coeficientes pelas porcentagens das categorias de cada uma das variáveis preditoras. Logo após, realizou-se a soma dos produtos e, ao final, obteve-se valor estimado de consumo de alimentos ultraprocessados do município A.


$$\text{\%}AUP\;estimado=constante\;+\left(\beta^{*}\text{\%}de\;mulheres\;\right)+\left(\beta^{*}\text{\%}de\;15\;a\;19\;anos\;\right)+\ldots$$

AUP = alimentos ultraprocessados.

β = coeficiente linear

Considerando as mudanças no perfil sociodemográfico da população brasileira entre 2010 e 2018, tais como o aumento da renda e da escolaridade, era de se esperar diferenças entre os valores diretamente mensurados pela POF 2017–2018 e as estimativas geradas a partir das variáveis da amostra do Censo 2010. Para corrigir essa diferença, foram aplicados fatores de correção nas estimativas de cada um dos 5.570 municípios. Esses fatores foram calculados utilizando a razão entre a média detectada diretamente pela POF 2017-2018 e o valor estimado a partir da amostra do Censo 2010 para cada uma das capitais brasileiras e do DF. O fator de correção de cada capital e do DF foi então aplicado a todos os municípios de sua respectiva UF, pressupondo uma diferença homogênea dentro da UF. As capitais brasileiras e o DF são os únicos que possuem, individualmente, representatividade populacional garantida pela amostragem da POF, possibilitando essa comparação e geração do fator de correção.

Após o Censo de 2010, o Brasil criou cinco municípios, os quais não possuem dados socioeconômicos disponíveis: Balneário Rincão (SC), Mojuí dos Campos (PA), Pinto Bandeira (RS), Paraíso das Águas (MS) e Pescaria Brava (SC). Para esses, assumimos a estimativa da participação calórica de alimentos ultraprocessados dos municípios a que eles pertenciam em 2010, a saber: Içara (SC), Santarém (PA), Bento Gonçalves (RS), Costa Rica (MS) e Laguna (SC), respectivamente.

As análises estatísticas foram realizadas com o *software* Stata 16.1. As estimativas de participação calórica de alimentos ultraprocessados foram apresentadas em um mapa de calor. A tonalidade do gráfico aumenta progressivamente com o aumento da categoria de participação de alimentos ultraprocessados. Utilizou-se o *software* QGIS e a Malha Municipal Digital do Brasil para a elaboração do mapa.

## RESULTADOS

A Tabela 1 apresenta a distribuição (frequências) das variáveis sociodemográficas e as médias de participação de alimentos ultraprocessados no total de calorias consumidas pela população brasileira com >10 anos, segundo a POF 2017-2018.

**Tabela 1:** Participação de alimentos ultraprocessados no total de calorias consumidas pela população brasileira com >10 anos ou mais de idade, segundo variáveis sociodemográficas. POF 2017–2018.

Característica		%	% de participação calórica de alimentos ultraprocessados		
			Média	IC95%	
Brasil		100	20,2	19,8	20,6
Sexo					
	Masculino	47,9	19,6	19,1	20,1
	Feminino	52,1	20,6	20,2	21,1
Faixa-etária (em anos)					
	10–14	8,5	27,5	26,7	28,4
	15–19	9,3	26,2	24,7	27,7
	20–24	8,5	24,1	23,1	25,2
	25–29	8.38	22,9	21,8	24,0
	30–34	8.56	20,5	19,5	21,5
	35–39	9,1	19,6	18,6	20,7
	40–44	8,0	17,5	16,7	18,3
	45–49	7,7	18,3	17,3	19,2
	50–54	7,5	16,8	15,9	17,6
	55–59	6,7	15,9	15,2	16,6
	60–64	5,6	15,3	14,6	16,0
	65–69	4,3	15,8	14,8	16,8
	> 70	7,7	15,3	14,6	16,0
Raça/cor autodeclarada					
	Branca	43,1	21,9	21,4	22,4
	Preta	10,8	18,8	17,8	19,8
	Amarela	0,7	23,5	20,1	26,8
	Parda	44,9	18,8	18,2	19,3
	Indígena	0,4	21,5	17,6	25,5
	Sem declaração	0,1	25,7	13,4	38,0
Escolaridade					
	Até o fundamental incompleto	41,9	17,2	16,8	17,5
	Fundamental completo a médio incompleto	16,8	20,7	19,9	21,4
	Médio completo a superior incompleto	30,7	22,5	21,7	23,3
	Superior completo	10,6	24,3	23,4	25,2
Renda *per capita* (salário mínimo)					
	≤ 1/4	4,9	15,8	14,7	16,9
	> 1/4–1/2	12,3	16,8	16,0	17,6
	> 1/2–1	24,3	18,4	17,9	19,0
	> 1–2	31,6	20,8	19,9	21,8
	> 2–3	11,8	21,8	29,9	22,7
	> 3–5	8,2	23,1	22,0	24,2
	> 5	6,9	25,9	24,5	27,4
Situação de domicílio					
	Urbano	85,5	21,2	20,7	21,7
	Rural	14,5	14,1	13,6	14,5
Situação do município					
	Capital	23,9	23,0	21,7	24,2
	Região Metropolitana	15,3	22,0	21,0	23,0
	Resto	60,8	18,6	18,2	19,0
Unidade da Federação					
	Rondônia	0,8	14,2	12,9	15,4
	Acre	0,4	16,7	14,7	18,6
	Amazonas	1,8	16,8	15,7	18,0
	Roraima	0,2	16,7	15,0	18,4
	Pará	3,9	18,8	17,4	20,3
	Amapá	0,4	21,3	19,7	22,8
	Tocantins	0,7	12,9	11,1	14,7
	Maranhão	3,3	13,1	12,1	14,0
	Piauí	1,6	11,5	10,8	12,3
	Ceará	4,3	17,8	16,6	19,0
	Rio Grande do Norte	1,7	18,7	17,6	19,9
	Paraíba	0,2	16,1	15,0	17,2
	Pernambuco	0,5	19,3	18,4	20,2
	Alagoas	1,6	16,6	15,4	17,9
	Sergipe	0,1	18,4	16,9	19,9
	Bahia	7,1	19,4	18,4	20,4
	Minas Gerais	10,2	19,5	18,6	20,3
	Espírito Santo	1,9	16,4	15,3	17,4
	Rio de Janeiro	8,4	21,1	20,1	22,2
	São Paulo	22,1	23,5	22,0	25,0
	Paraná	5,5	20,7	19,4	22,1
	Santa Catarina	3,4	23,9	22,6	25,1
	Rio Grande do Sul	5,6	24,8	23,7	26,0
	Mato Grosso do Sul	1,3	19,6	18,3	20,9
	Mato Grosso	0,2	16,7	15,3	18,1
	Goiás	3,4	17,4	16,2	18,6
	Distrito Federal	1,4	22,5	21,0	23,9

Em 2017–2018, a frequência de mulheres era ligeiramente maior do que a de homens (52,1% *versus* 47,9%), 17,8% eram adolescentes e 17,6% eram idosos. A raça/cor parda concentrou a maior fração da população (44,9%) e 41,9% tinham escolaridade até o fundamental incompleto. A maior parte (55,9%) da população possuía renda *per capita* de meio a dois salários mínimos. Cerca de 85% residiam no meio urbano e a maior parte morava no estado de São Paulo (22,1%).

Em 2017–2018, a média de participação calórica de alimentos ultraprocessados no Brasil foi de 20,2% (intervalos de confiança de 95% - IC95% 19,8–20,6), com variação segundo características sociodemográficas. A média da participação calórica foi ligeiramente superior em mulheres em comparação com homens, diminuiu com o aumento da idade e aumentou conforme nível de escolaridade e renda. A média de participação calórica foi maior em indivíduos do meio urbano, em comparação com os do rural, e nas capitais em relação aos demais municípios. As médias de participação calórica foram mais altas no DF e nos estados do Sul e Sudeste, exceto no Espírito Santo. Por outro lado, os estados da região Norte e Nordeste, com exceção da Bahia, apresentaram as médias mais baixas (Tabela 1).

### Modelo de Predição da Participação Calórica de Alimentos Ultraprocessados

A Tabela 2 apresenta os coeficientes β e seus respectivos IC95% do modelo de regressão linear múltiplo, que testa a associação entre as variáveis preditoras e a participação calórica de alimentos ultraprocessados no total de calorias consumidas pela população brasileira com >10 anos. Todas as variáveis preditoras foram estatisticamente associadas com o desfecho (p < 0,001). As variáveis preditoras que apresentaram os maiores coeficientes β e, portanto, com maior poder explicativo, foram renda familiar *per capita* e idade. Indivíduos com renda familiar *per capita* >5 salários mínimos apresentaram uma média de participação calórica de alimentos ultraprocessados 8,4 pontos percentuais (p.p.) maior em comparação àqueles com renda familiar *per capita* menor ou igual a menos de 1/4 de salário-mínimo. Indivíduos com idade igual ou superior a 70 anos apresentaram uma média de participação calórica de alimentos ultraprocessados 15,8 p.p. menor em relação àqueles com idades entre 10 e 14 anos.

**Tabela 2:** Coeficientes β e seus respectivos intervalos de confiança de 95% do modelo de regressão linear, associando as variáveis preditoras e a participação calórica de alimentos ultraprocessados no total de calorias consumidas pela população brasileira com >10 anos ou mais de idade. POF 2017–2018.

Característica		% de participação calórica de alimentos ultraprocessados			
		β	IC95%		p-valor
Sexo					< 0,001
	Masculino	Ref.			
	Feminino	1,3	0,9	1,7	
Faixa-etária (em anos)					< 0,001
	10–14	Ref.			
	15–19	-2,9	-4,4	-1,4	
	20–24	-6,5	-7,8	-5,1	
	25–29	-8,4	-9,7	-7,1	
	30–34	-10,5	-11,6	-9,4	
	35–39	-11,4	-12,5	-10,3	
	40–44	-13,2	-14,3	-12,1	
	45–49	-12,8	-13,9	-11,6	
	50–54	-14,4	-15,5	-13,3	
	55–59	-15,5	-16,5	-14,4	
	60–64	-16,2	-17,2	-15,1	
	65–69	-15,5	-16,7	-14,2	
	> 70	-15,8	-16,9	-14,7	
Raça/cor autodeclarada					< 0,001
	Branca	Ref.			
	Preta	-0,9	-2,1	0,2	
	Amarela	0,3	-2,4	3,0	
	Parda	-0,8	-1,5	0,0	
	Indígena	1,7	-1,7	5,1	
	Sem declaração	4,4	-5,5	14,4	
Escolaridade					0,002
	Até o fundamental incompleto	Ref.			
	Fundamental completo a médio incompleto	0,8	0,0	1,5	
	Médio completo a superior incompleto	2,8	2,1	3,6	
	Superior completo	3,6	2,5	4,6	
Renda *per capita* (salário mínimo)					< 0,001
	≤ 1/4	Ref.			
	> 1/4–1/2	0,9	-0,4	2,2	
	> 1/2–1	2,6	1,3	3,8	
	> 1–2	5,0	3,7	6,4	
	> 2–3	5,6	4,2	7,0	
	> 3–5	6,2	4,5	7,9	
	> 5	8,4	6,4	10,3	
Situação de domicílio					< 0,001
	Urbano	Ref.			
	Rural	-3,4	-4,1	-2,8	
Situação do município					< 0,001
	Capital	Ref.			
	Região Metropolitana	-1,5	-3,1	0,2	
	Resto	-2,4	-3,8	-1,1	
Unidade da Federação					< 0,001
	Rondônia	Ref.			
	Acre	1,2	-1,1	3,4	
	Amazonas	0,9	-0,9	2,7	
	Roraima	0,3	-2,2	2,7	
	Pará	5,1	3,1	7,1	
	Amapá	5,0	3,0	7,1	
	Tocantins	-1,0	-3,2	1,2	
	Maranhão	0,3	-1,3	1,8	
	Piauí	-1,4	-3,0	0,1	
	Ceará	4,0	2,3	5,7	
	Rio Grande do Norte	4,4	2,7	6,1	
	Paraíba	2,9	1,2	4,6	
	Pernambuco	5,2	3,6	6,8	
	Alagoas	3,3	1,5	5,0	
	Sergipe	3,6	1,8	5,4	
	Bahia	5,7	4,1	7,3	
	Minas Gerais	4,8	3,3	6,4	
	Espírito Santo	1,6	-0,2	3,3	
	Rio de Janeiro	5,0	3,3	6,7	
	São Paulo	7,0	4,9	9,1	
	Paraná	5,1	3,3	6,9	
	Santa Catarina	8,5	6,6	10,4	
	Rio Grande do Sul	9,3	7,5	11,1	
	Mato Grosso do Sul	3,4	1,5	5,2	
	Mato Grosso	1,4	-0,5	3,2	
	Goiás	1,7	-0,1	3,5	
	Distrito Federal	3,2	1,2	5,3	

Os resíduos do modelo apresentaram distribuição próxima da normal (Figura 1), indicando a sua validade para capturar adequadamente a relação entre as variáveis preditoras e o desfecho. Além disso, a concordância entre a média da participação calórica de alimentos ultraprocessados predita pelo modelo e a detectada diretamente na POF 2017/2018 para as 26 capitais brasileiras e no DF foi considerada satisfatória, com um CCC de 0,87 (concordância quase perfeita) (Figura 2).

**Figura 1:** Distribuição dos resíduos do modelo de regressão linear múltiplo associando as variáveis preditoras e a participação calórica de alimentos ultraprocessados no total de calorias consumidas pela população brasileira com >10 anos ou mais de idade. A distribuição normal indica um modelo válido. POF 2017/2018.

**Figura 2:** Concordância entre a média da participação calórica de alimentos ultraprocessados predita pelo modelo ajustado e a detectada diretamente na POF 2017/2018.

CCC: coeficiente de correlação-concordância de Lin.

### Estimativas de Participação Calórica de Alimentos Ultraprocessados nos 5.570 Municípios do Brasil

As estimativas da participação calórica de alimentos ultraprocessados para cada um dos 5.570 municípios estão ilustradas no mapa de calor (Figura 3)^a^. Foi observada uma heterogeneidade nas estimativas de participação calórica de alimentos ultraprocessados no país. O intervalo de variação abrangeu municípios com participação calórica tão baixa quanto cerca de 6% (5,75% em Aroeiras do Itaim, Piauí; 5,83% em Dois Irmãos do Tocantins e 5,87% em Monte Santo do Tocantins, ambos em Tocantins) e tão alta quanto cerca de 30% (27,8% em Balneário Camboriú, 28,3% em São José e 30,5% em Florianópolis, todos em Santa Catarina). As capitais apresentam maiores estimativas de participação calórica de alimentos ultraprocessados em relação aos demais municípios de seus respectivos estados.

**Figura 3:** Distribuição espacial das estimativas de participação calórica de alimentos ultraprocessados nos municípios brasileiros^a^.

As estimativas de participação calórica de alimentos ultraprocessados foram mais altas nos municípios da região Sul, especialmente naqueles do estado de Santa Catarina, onde todos os municípios apresentaram estimativas acima de 20%. No Paraná, as estimativas variaram de 16% no município de Antônio Olinto a 26,3% em Curitiba. No Rio Grande do Sul, as estimativas variaram de 17,6% no município de Coqueiro Baixo a 26,6% em Porto Alegre.

No Sudeste, os municípios do estado de São Paulo apresentaram as maiores estimativas, com a capital chegando a 25,5%. Por outro lado, os municípios do estado do Espírito Santo apresentaram as menores estimativas da região, com a capital Vitória apresentando 16,7%. O estado de Minas Gerais destacou-se como um dos estados com as estimativas municipais mais heterogêneas na região, com Frei Lagonegro atingindo 13,8% e, no outro extremo, os municípios Nova Lima e Belo Horizonte atingindo 20,9% e 22,8%, respectivamente. Os municípios do estado do Rio de Janeiro apresentaram estimativas variando de 15,2% para São José de Ubá a 22,4% na capital.

No Centro-Oeste, os municípios de Goiás apresentaram as menores estimativas da região, sendo a mais alta registrada na capital Goiânia (19,4%). Brasília foi o município com maior média de participação calórica de alimentos ultraprocessados na região, atingindo 22,6%. Mato Grosso destacou-se como o estado com maior heterogeneidade nas estimativas dos municípios da região, variando de 13,0% em Barão de Melgaço e Porto Estrela a cerca de 18,0% em Campo Novo dos Parecis, Várzea Grande e Cuiabá.

No Nordeste, os estados cujos municípios apresentaram as menores estimativas foram, em ordem crescente, Piauí, Maranhão e Paraíba. O estado da Bahia destacou-se por apresentar municípios com as maiores estimativas da região, com todos acima de 16,0% e a capital Salvador alcançando 25,7%.

Os municípios do estado de Tocantins apresentaram as menores estimativas de participação calórica de alimentos ultraprocessados da região Norte, com a mais alta sendo na capital Palmas, que alcançou 12,2%. Os estados de Roraima e Rondônia apresentaram estimativas intermediárias na região. O município de Cantá, em Roraima, apresentou estimativa de 9,25% e o município de Novo Horizonte do Oeste, em Rondônia, de 9,46%, enquanto as capitais apresentaram estimativas de 15,6% e 16,6%, respectivamente. Os municípios dos estados do Pará e Amapá, por sua vez, apresentaram as maiores estimativas da região.

## DISCUSSÃO

Este estudo, utilizando um modelo estatístico de predição construído a partir de dados representativos de um inquérito alimentar, estimou a participação calórica de alimentos ultraprocessados dos 5.570 municípios brasileiros, que variou de 5,7% a 30,5%. O modelo de predição foi considerado válido para estimar a participação calórica dos alimentos ultraprocessados.

A grande variação nas estimativas obtidas neste estudo reflete a distribuição desigual das características sociodemográficas entre os municípios. Por exemplo, todos os municípios com mais de 15,0% da população com renda *per capita* igual ou superior a cinco salários mínimos apresentaram estimativas de participação calórica de alimentos ultraprocessados acima de 20,0%, com exceção de Vitória (ES). Em contrapartida, a grande maioria dos municípios em que 0% da população tem renda *per capita* superior a cinco salários mínimos apresentou estimativas bem inferiores a esse valor.

Além disso, ter maior proporção de moradores em áreas urbanas também se mostrou determinante na estimativa de participação calórica de alimentos ultraprocessados nos municípios brasileiros. Os estados do Maranhão, Piauí e Tocantins, que possuem as maiores proporções de pessoas morando em área rural do país, apresentaram as estimativas de participação calórica de alimentos ultraprocessados mais baixas. Por outro lado, São Paulo, um dos estados com a maior proporção de moradores na área urbana do país, apresentou estimativas municipais acima de 18,0%. Além disso, todas as 26 capitais e o DF apresentaram estimativas mais elevadas em comparação com os demais municípios de seus respectivos estados.

É importante pontuar que, mesmo que se considere positivo que alguns municípios possuam baixas estimativas de participação calórica de alimentos ultraprocessados, isso não implica necessariamente melhor desempenho em outros indicadores de qualidade da alimentação. Há evidências de que domicílios de baixa renda e das regiões rurais do país, por exemplo, possuem alto consumo de alimentos *in natura* ou minimamente processados e ingredientes culinários, mas que esse consumo é marcado por alimentos básicos, como arroz, feijão, café, açúcar, farinhas e carnes, e que o consumo de outros alimentos saudáveis, como frutas, legumes e verduras, além da diversidade alimentar, é muito aquém do recomendado^12^.

Estudo anterior, utilizando dados de disponibilidade domiciliar de alimentos demonstrou que a aquisição de alimentos ultraprocessados aumentou de 14,3% das calorias totais, em 2002-2003, até 19,4%, em 2017-2018^13^. De forma similar, Louzada e colaboradores^8^, utilizando dados de consumo alimentar individual, observaram um aumento do consumo efetivo de alimentos ultraprocessados entre o período de 2008-2009 a 2017-2018. Fica nítido que o aumento de consumo dos alimentos ultraprocessados foi mais acentuado nas regiões Norte e Nordeste do país, além das regiões rurais e naqueles com menor renda *per capita*. Desta forma, os resultados indicam que aqueles municípios com o menor consumo estimado no presente estudo são, muito provavelmente, aqueles que também estão apresentando um maior aumento no seu consumo, indicando que as estimativas podem ser diferentes na ocasião de uma atualização de uma nova POF.

A estimativa de participação calórica de alimentos ultraprocessados é relevante e pode apoiar diversas iniciativas, como a investigação de fatores ambientais relacionados à produção, oferta e disponibilidade de alimentos ultraprocessados que influenciam a realidade local, o desenvolvimento de políticas públicas específicas e direcionadas e o monitoramento do impacto dessas políticas por meio da realização contínua de estimativas baseadas em futuras POF. Tais ações podem ser fundamentais para promover uma alimentação mais saudável e sustentável de maneira mais equitativa.

A adesão municipal ao Sistema Nacional de Segurança Alimentar e Nutricional (Sisan) permite o acesso a recursos do Governo Federal para políticas e programas a nível municipal, como o Programa de Cozinhas Solidárias, que tem como objetivo fornecer o acesso à alimentação saudável, respeitados os preceitos do Guia Alimentar para a População Brasileira. Outro exemplo é o Programa Restaurante Popular, um equipamento social integrante da estrutura operacional do Sisan, que visa ampliar a oferta de refeições nutricionalmente adequadas a preços acessíveis para populações em situação de vulnerabilidade social. No estado de São Paulo, o programa Bom Prato destaca-se como um dos maiores do país. Ações locais, como as leis municipais do Rio de Janeiro e de Niterói que proíbem a comercialização de alimentos ultraprocessados em cantinas de escolas públicas e privadas, são exemplos de iniciativas que buscam reduzir o consumo desses produtos entre crianças e adolescentes^14,15^.

Recentemente, o Ministério do Desenvolvimento e Assistência Social, Família e Combate à Fome lançou um plano estratégico que identifica 60 cidades prioritárias para a implementação da Estratégia Nacional de Segurança Alimentar e Nutricional nas Cidades, denominada Alimenta Cidades^16^. Essa iniciativa busca ampliar a produção, o acesso, a disponibilidade e o consumo de alimentos saudáveis para populações em situação de vulnerabilidade social. Entre as cidades priorizadas, destacam-se todas as capitais do país, que apresentaram estimativas elevadas de consumo de alimentos ultraprocessados em comparação aos demais municípios de seus estados.

O estudo apresenta como principal limitação a diferença temporal entre a POF 2017-2018 e o Censo Demográfico de 2010. Embora um novo Censo tenha sido realizado, em 2022, seus dados (como de renda) não estão disponíveis para consulta. No entanto, para mitigar essa limitação, fatores de correção para cada uma das 26 capitais e o DF foram calculados e aplicados nos municípios de suas respectivas UF. Além disso, o módulo de consumo alimentar da POF 2017-2018 não incluiu pessoas com >10 anos, o que determina que os resultados do presente estudo se aplicam apenas à população brasileira de adolescentes e adultos. Como fortalezas, pode-se citar que o modelo de predição criado para obter as estimativas de consumo de alimentos ultraprocessados nos municípios apresentou resultados satisfatórios de adequabilidade, ao apresentar um resíduo com distribuição próxima da normal e uma concordância alta entre a média de participação calórica predita pelo modelo e a detectada diretamente na POF 2017-2018 para as capitais e o DF. Além disso, o estudo, de maneira pioneira, gerou estimativas municipais de participação calórica de alimentos ultraprocessados, uma informação valiosa para os gestores municipais.

Em conclusão, este estudo gerou estimativas de participação calórica de alimentos ultraprocessados nos municípios brasileiros. Foi possível observar uma heterogeneidade nas estimativas municipais, que são reflexos da desigualdade sociodemográfica da população brasileira. As estimativas geradas podem contribuir para o monitoramento do consumo alimentar de ultraprocessados a nível municipal e fortalecer e subsidiar a criação de políticas públicas focadas na promoção da alimentação saudável.

## Footnotes

a Também disponíveis em: https://doi.org/10.17605/OSF.IO/ZM9CR.
